# Primary human intestinal organoids with biallelic UNC45A variants suggest role of cystic fibrosis transmembrane conductance regulator in pathogenesis of UNC45A‐related intestinal disorder

**DOI:** 10.1002/jpn3.70230

**Published:** 2025-10-17

**Authors:** April Rose Foster, Natasha G., Rebecca Harris, Matthias Zilbauer, Alexander Ross

**Affiliations:** ^1^ Wellcome Sanger Institute, Wellcome Genome Campus Cambridge UK; ^2^ University Department of Pediatrics University of Cambridge Cambridge UK; ^3^ Milner Therapeutics Institute, Jeffrey Cheah Biomedical Centre University of Cambridge Cambridge UK; ^4^ Wellcome‐MRC Cambridge Stem Cell Institute University of Cambridge Cambridge UK; ^5^ Department of Paediatric Gastroenterology, Hepatology and Nutrition, Addenbrooke's Hospital Cambridge University Hospitals NHS Foundation Trust Cambridge UK; ^6^ Wessex Clinical Genetics Service University Hospital Southampton NHS Foundation Trust Southampton UK; ^7^ Institute of Developmental Sciences University of Southampton Southampton UK

**Keywords:** congenital, diarrhoea, forskolin, genetics, intestine

## Abstract

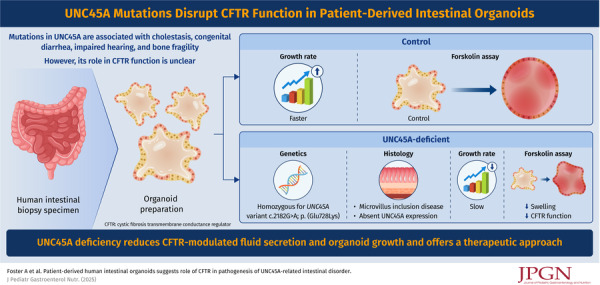

## 1 INTRODUCTION

Biallelic loss‐of‐function variants in *UNC45A* have recently been described as being associated with a syndrome of cholestasis, congenital diarrhea, impaired hearing, and bone fragility.^1^ UNC45A is a molecular chaperone involved in the folding and functional maintenance of myosins, including MYO5B, a protein crucial for intracellular trafficking. Cystic fibrosis transmembrane conductance regulator (CFTR) is a chloride channel in the apical membrane of enterocytes and is important for the maintenance of fluid homeostasis. MYO5B helps facilitate the trafficking of CFTR to the apical membrane and impairment in MYO5B function could disrupt this process, leading to altered CFTR localization and/or activity. When UNC45A is nonfunctional, MYO5B function is thought to be impaired, leading to defective trafficking of apical proteins (potentially including CFTR) and the formation of microvillus inclusions.

While CFTR is an important regulator of fluid movement in tissues directly involved in UNC45A‐related disorder, including cholangiocytes, the inner ear, and the intestinal epithelium, there is currently no evidence that links CFTR dysfunction to disease pathogenesis.

Duclaux‐Loras et al.^2^ recently characterized six patients across five families with UNC45A‐deficiency and utilized a forskolin swelling assay (FSA) to assess for the potential involvement of CFTR in *UNC45A*‐dependent disease. The FSA is a well‐described method to assess CFTR function in intestinal organoids. Forskolin is a small molecule that increases the activity of intracellular adenylyl cyclase, which results in increased cyclic adenosine monophosphate (cAMP). When there is increased cAMP, protein kinase A (PKA) becomes activated, which phosphorylates and activates CFTR. When CFTR is functional, this leads to chloride ion transport across the apical cell membrane and therefore organoid swelling. The rate of organoid swelling should be proportional to CFTR activity, when epithelial barrier function is intact.^3^

This previous work derived induced pluripotent stem cells (iPSC) from blood and differentiated these iPSC to intestinal epithelium‐like organoids. Subsequently, they demonstrated that addition of 10 µM forskolin to both the patient‐derived and control organoid lines produced the same response, with no statistically significant difference between the two groups, leading the authors to conclude that CFTR function was not affected by loss of UNC45A.

In this study, we provide additional functional data generated using mucosa‐derived intestinal epithelial organoids obtained from a patient with biallelic pathogenic variants in *UNC45A*. We have previously reported on the distinct epigenetic and transcriptional differences between mucosa‐derived (i.e., adult stem cell) and iPSC‐derived intestinal epithelial organoids,^4^ with the latter demonstrating substantial differences in gut segment‐specific molecular signatures when compared to the primary epithelium. Thus, we hypothesized that the absence of any differences in organoid swelling identified by Duclaux‐Loras et al. may represent the limitations of the model used. The key outcome measures of this study were organoid growth and organoid swelling after application of the FSA; both of which were reduced in the *UNC45A‐*related disorder patient‐derived organoids.

## 2 METHODS

### 2.1 Ethics statement

All experimental protocols were approved by National Research Ethics Service (NRES), East of England under the REC number 12/EE/0482. All methods were carried out in accordance with the guidelines and regulations set out by these documents and informed consent was obtained from the legal guardian of each participant.

### 2.2 Study details

#### 2.2.1 Derivation of intestinal organoids

Human duodenal intestinal biopsy specimens were obtained from a pediatric patient with suspected *UNC45A*‐related disorder and three age‐matched control individuals. Primary human intestinal epithelial organoids were subsequently generated from biopsy specimens through crypt isolation and resuspension in Matrigel® (Corning) and addition of intestinal organoid culture medium as described previously.^5^ The culture medium was replaced with fresh medium every 48–72 h and intestinal organoids were passaged every 5–7 days via mechanical disruption with a P1000 pipette.

#### 2.2.2 Assessment of growth characteristics of intestinal organoids

Three‐dimensional organoids were visualised in two dimensions using light microscopy and every 6 h in an automated manner using the Incucyte SX5 Organoid Module. Organoid growth was analyzed using the Incucyte Organoid Analysis Software Module as an estimation based on organoid size normalized to timepoint 0 h (time of plating).

#### 2.2.3 Forskolin treatment of intestinal organoid

Intestinal organoids were passaged after 7 days from establishment and resuspended in Matrigel® in a 96‐well plate. Dilution of organoids was carried out to ensure approximately 10–20 organoids per well. Culture medium was replaced every 48 h for 4 days. On Day 5 of culture, the forskolin assay was started by addition of either vehicle control medium or 5 µM forskolin in intestinal organoid media (please note, Duclaux‐Loras et al.^2^ used a concentration of 10 µM forskolin). The plate was imaged using the Incucyte SX5 over a 60‐h period at 2‐h intervals. Images captured were imaged using the Sartorius Incucyte Organoid Module to assess organoid size normalized to timepoint 0 h. This was carried out on organoids derived from three control patients and one UNC45A‐deficient patient with three technical replicates carried out per organoid line. Analysis was performed using GraphPad® prism software; one‐way analysis of variance (ANOVA) test applied, with a significance threshold *p* < 0.05. The data are presented as mean ± 1 standard deviation.

#### 2.2.4 UNC45A sequencing

Sequencing of the affected individual was carried out by Cambridge University Hospitals Genomics Laboratories. The Laboratory is accredited by The United Kingdom Accreditation Service (UKAS) to the recognized international standard ISO 15189:2012. Mapping was carried out using the GRCh38 reference genome, with variant co‐ordinates: 15:90950262. In vitro experimentation carried out from October 2021 to March 2022.

## 3 RESULTS

**Figure 1:**
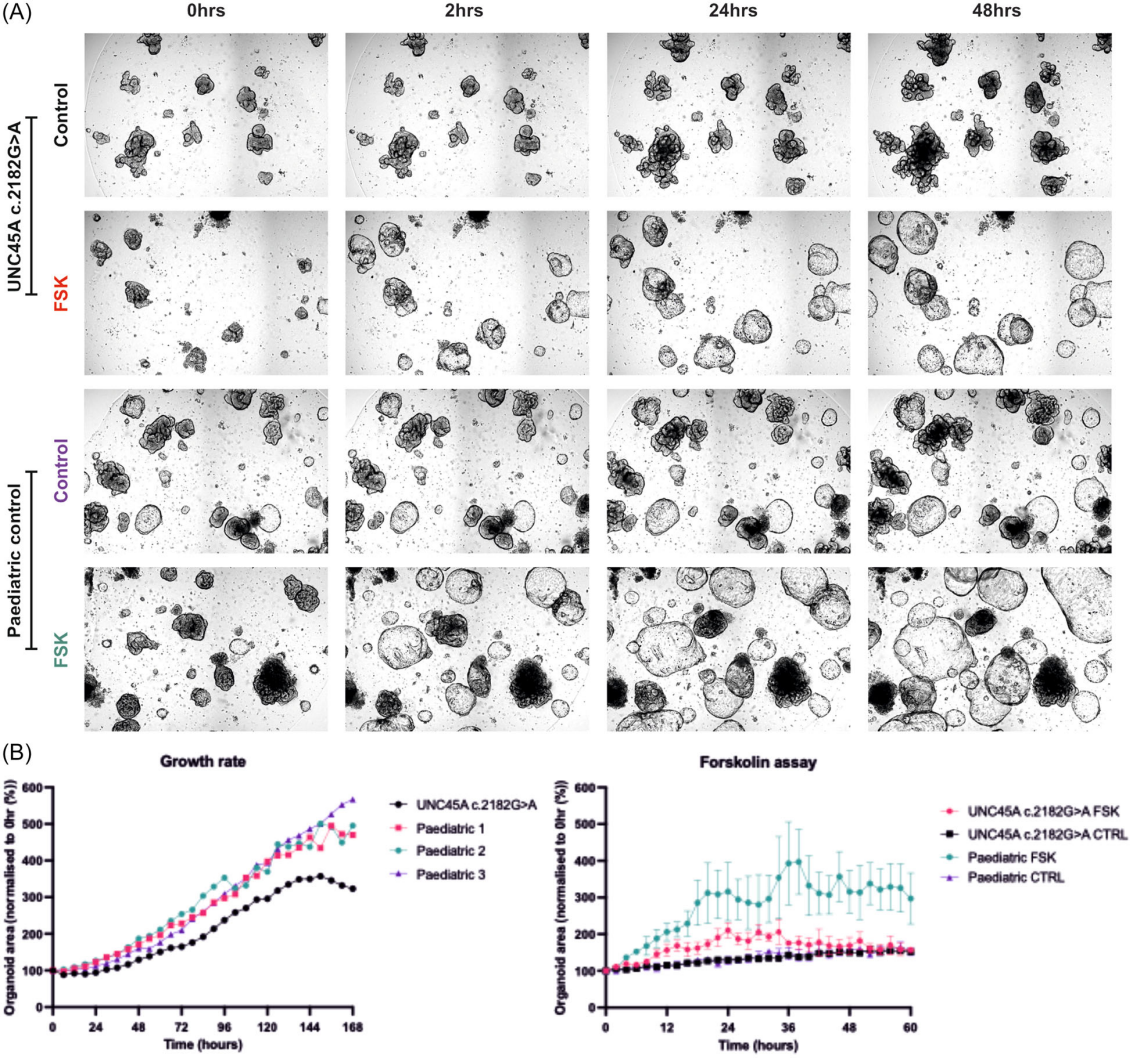
(A) Brightfield of UNC45A‐deficient and control organoid lines before (0‐h), after 2‐, 24‐, and 48‐h forskolin treatment. (B) Organoid growth over 7‐days of organoids derived from an individual with *UNC45A* homozygous variants compared to *n* = 3 controls organoid lines (each control displayed as separate line and numbered in legend) and (C) forskolin assay in UNC45A‐deficient and control organoids (*n* = 3, mean represented). FSK, forskolin.

Primary intestinal organoids were derived from an individual with congenital diarrhea, as described above. Subsequent histology demonstrated characteristic features of microvillus inclusion disease and lack of *UNC45A*. Sequencing of *UNC45A* demonstrated the individual was homozygous for the *UNC45A* variant c.2182 G > A; p. (Glu728Lys). This missense variant has previously been described in an individual with UNC45A deficiency.^2^ We then compared the UNC45A‐deficient organoids to control organoid lines (*n* = 3). We exposed these organoids to 5 µM forskolin and imaged them every 2 h over a 60‐h period using the Incucyte SX5. Our findings demonstrated a significant difference in the rate and extent of organoid swelling following forskolin stimulation over longer time points, with a difference in organoid size becoming significant from 12 h onwards (Figure 1A,C). Our data also suggest that UNC45A‐deficient organoids have a slower rate of growth compared to control organoids (Figure 1B).

## 4 DISCUSSION

These data suggest that there may be an important role of CFTR in the pathogenesis of *UNC45A*‐related intestinal disorder, and that experimental design of functional organoid experiments can be crucial, particularly when considering possible null effects. Understanding the mechanism of intestinal pathology is of critical importance in developing appropriate management strategies related to fluid regulation of the gut, and for development of potential therapeutic interventions.

We previously demonstrated that there are significant differences in morphology, transcriptomic profile and methylome profile between iPSC‐derived organoids and primary intestinal organoids, and that such large‐scale differences are highly likely to impact on respective organoid function.^4^

The reduced organoid growth observed in organoids derived from the individuals with *UNC45A*‐related disorder may indicate an alteration in the activity of the stem cell niche that may be relevant to the disorder and therefore assessment of cell division dynamics could represent an interesting area of exploration in future studies.

Organoid swelling can be influenced by both the rate of solute transfer across the apical membrane to the organoid lumen and by paracellular loss of intralumenal contents due to increased epithelial barrier permeability. Thus, further work examining the intracellular distribution of CFTR protein and direct measurement of epithelial barrier function could be highly informative. If CFTR were fully elucidated as a potential therapeutic target, significant challenges would include identifying which agents could restore CFTR to baseline functioning and how to deliver such a thereapeutic agent to a tissue with a surface area as large as the human intestinal epithelium.

These data could lend further support to previous work performed by Lechuga et al., where depletion of UNC45A resulted in increased paracellular permeability and impaired epithelial barrier function.^6^ Importantly, further studies that utilise organoids from other individuals with UNC45‐related disorder, beyond the single individual from this study, are required to further examine the pervasiveness of this response. Additionally, direct assays of membrane permeability and CFTR localisation could be key to investigating the pathogenic mechanism of this disorder. We also suggest that future experiments using the forskolin assay to investigate *UNC45A*‐related intestinal disorder should be optimized for dose and duration of treatment, and consideration given to the potential impact of utilizing primary gut‐derived organoids as compared to blood‐derived iPSC organoids.

## 5 CONCLUSION

Our findings suggest that UNC45A deficiency impairs CFTR‐related function and epithelial growth in primary intestinal organoids, contrasting with previous results from iPSC‐derived models. This highlights the importance of using tissue‐specific organoids in functional studies and supports a potential role for CFTR dysfunction in UNC45A‐related disease. Further studies are needed to confirm these findings and explore therapeutic implications.

## CONFLICT OF INTEREST STATEMENT

There is no conflict of interest for this study.

## Supporting information

JPGNJ 229 3 Foster Infographics Sep 10 2025.

## References

[1] Esteve C , Francescatto L , Tan PL , et al. Loss‐of‐function mutations in UNC45A cause a syndrome associating cholestasis, diarrhea, impaired hearing, and bone fragility. Am J Hum Genet. 2018;102(3):364‐374. 10.1016/j.ajhg.2018.01.009 29429573 PMC5985364

[2] Duclaux‐Loras R , Lebreton C , Berthelet J , et al. UNC45A deficiency causes microvillus inclusion disease‐like phenotype by impairing myosin VB‐dependent apical trafficking. J Clin Invest. 2022;132(10):e154997. 10.1172/JCI154997 35575086 PMC9106349

[3] Dekkers JF , Wiegerinck CL , de Jonge HR , et al. A functional CFTR assay using primary cystic fibrosis intestinal organoids. Nature Med. 2013;19(7):939‐945. 10.1038/nm.3201 23727931

[4] Kraiczy J , Ross ADB , Forbester JL , Dougan G , Vallier L , Zilbauer M . Genome‐wide epigenetic and transcriptomic characterization of human‐induced pluripotent stem cell‐derived intestinal epithelial organoids. Cell Mol Gastroenterol Hepatol. 2019;7(2):285‐288. 30704978 10.1016/j.jcmgh.2018.10.008 PMC6354438

[5] Howell KJ , Kraiczy J , Nayak KM , et al. DNA methylation and transcription patterns in intestinal epithelial cells from pediatric patients with inflammatory bowel diseases differentiate disease subtypes and associate with outcome. Gastroenterology. 2018;154:585‐598. 29031501 10.1053/j.gastro.2017.10.007 PMC6381389

[6] Lechuga S , Cartagena‐Rivera AX , Khan A , et al. A myosin chaperone, UNC‐45A, is a novel regulator of intestinal epithelial barrier integrity and repair. FASEB J. 2022;36(5):e22290. 35344227 10.1096/fj.202200154R PMC9044500

