# Supplementary figures and images for: Primary human intestinal organoids with biallelic UNC45A variants suggest role of cystic fibrosis transmembrane conductance regulator in pathogenesis of UNC45A‐related intestinal disorder
Source: J Pediatr Gastroenterol Nutr. 2025 Oct 17;82(1):70–3. doi: 10.1002/jpn3.70230 (PMC12780477; doi:10.1002/jpn3.70230)

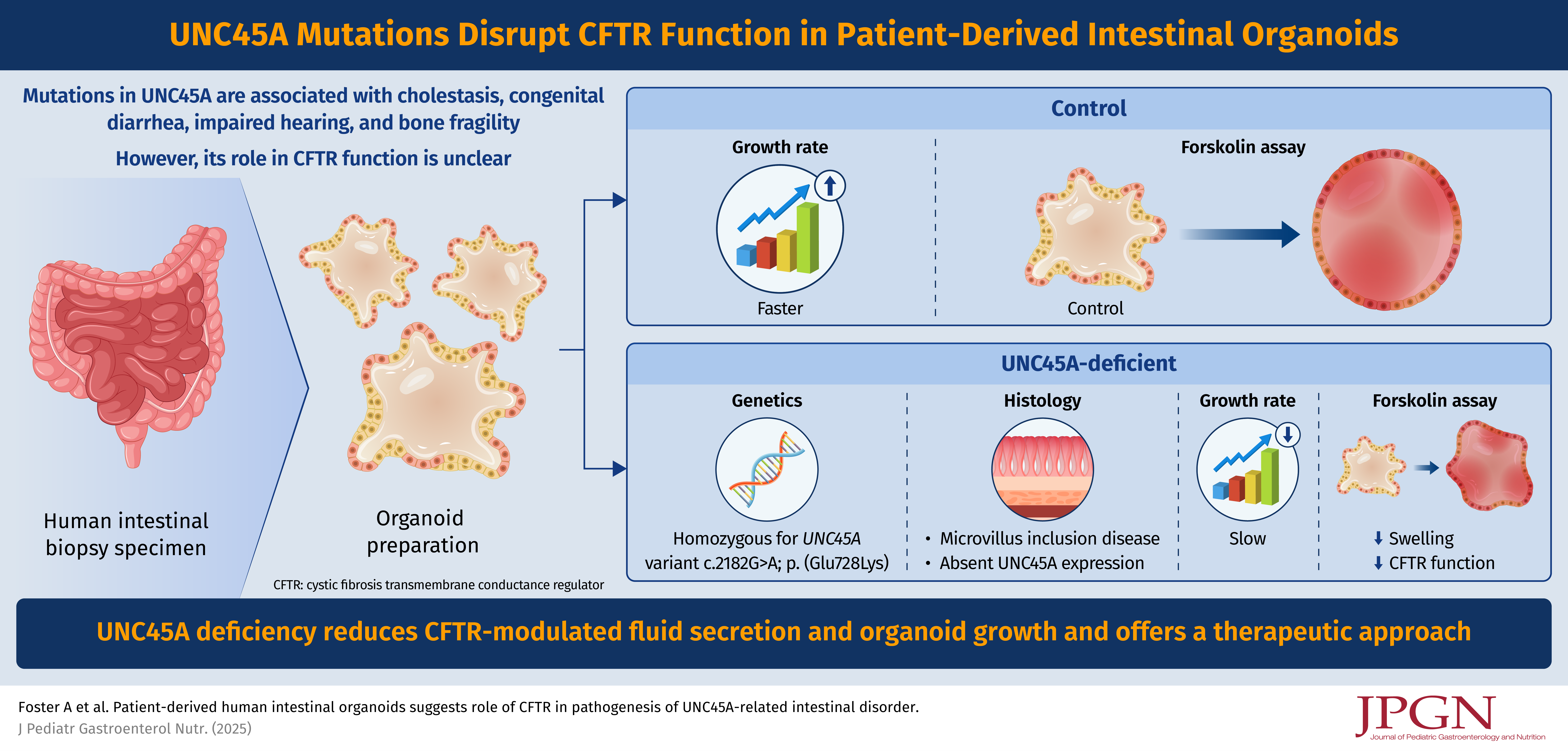

Supplement: Supplementary file 1 — JPGNJ 229 3 Foster Infographics Sep 10 2025. [file JPN3-82-70-s001.png]
